# IL-1α and IL-1β-producing macrophages populate lung tumor lesions in mice

**DOI:** 10.18632/oncotarget.11276

**Published:** 2016-08-12

**Authors:** Michela Terlizzi, Chiara Colarusso, Ada Popolo, Aldo Pinto, Rosalinda Sorrentino

**Affiliations:** ^1^ Department of Pharmacy (DIFARMA), University of Salerno, Salerno, Fisciano, 84084, Italy; ^2^ PhD Program in Drug Discovery and Development, University of Salerno, Salerno, Fisciano, 84084, Italy

**Keywords:** NLRP3 inflammasome, lung cancer, IL-1α, IL-1β, tumor-associated macrophages

## Abstract

Macrophages highly populate tumour microenvironment and are referred to as tumor-associated macrophages (TAMs). The inflammasome is a multiprotein complex responsible of IL-1 like cytokines release, which biology has been widely studied by using bone-marrow-derived macrophages to mimic a physiological and/or host defense condition. To understand the role of this complex in lung tumor-associated macrophages (TAMs), we isolated and cultured broncho-alveolar lavage (BAL)-derived cells of lung tumor-bearing mice. The stimulation of lung TAMs with LPS+ATP increased the release of IL-1β. The inhibition of NLRP3 by means of glybenclamide significantly reduced IL-1β release. Similarly, C3H-derived, caspase-1 ko and caspase-11 ko TAMs released significantly reduced levels of IL-1β. Moreover, the stimulation of lung TAMs with the sole LPS induced a significant release of IL-1α, which was significantly reduced after caspase-1 pharmacological inhibition, and in TAMs genetically lacking caspase-1 and caspase-11. The inhibition of calpain I/II by means of MDL28170 did not alter IL-1α release after LPS treatment of lung TAMs. To note, the inoculation of LPS-treated bone marrow-derived macrophages into carcinogen-exposed mice increased lung tumor formation. In contrast, the depletion of TAMs by means of clodronate liposomes reduced lung tumorigenesis, associated to lower *in vivo* release of IL-1α and IL-1β.

In conclusion, our data imply lung tumor lesions are populated by macrophages which pro-tumor activity is regulated by the activation of the NLRP3 inflammasome that leads to the release of IL-1α and IL-1β in a caspase-11/caspase-1-dependent manner.

## INTRODUCTION

Tumor microenvironment is a complex scenario in which immune cells play a critical role because they can interfere with tumor cell survival or death [1]. Macrophages highly populate tumour microenvironment and are referred to as tumor-associated macrophages (TAMs) [2]. TAMs can be classically active (M1) or alternatively activated macrophages (M2). In the first case, macrophages highly produce pro-inflammatory cytokines, highly express MHC molecules and have a potent tumoricidal activity [3]. In contrast, the M2 phenotype is characterized by the production of high amounts of IL-10 and TGF-β, immunosuppressive cytokines, involved in anti-inflammatory activities and tissue repair functions [3]. Clinical and experimental evidence have shown that cancer tissues with high infiltration of TAMs in their M2 phenotype are associated with poor patient prognosis, angiogenesis and resistance to therapies [4], whereas the M1 phenotype can favor tumor regression [5]. In particular, a strong association between poor prognosis and macrophage infiltration has been observed in non-small cell lung cancer (NSCLC) patients [6], evidence that imply the relevant role of these cells during lung carcinogenesis and tumor progression. Another important epidemiological data correlates high serum and tissue levels of IL-1β to the lung malignancy with low-rate survival from time of diagnosis [7].

Recent studies identified the multimeric complex of the inflammasome as responsible for IL-1β/IL-18 synthesis/release from bone-marrow-derived macrophages (BMDM) under physiological conditions [7]. However, the role of the inflammasome in cancer is still controversial [8]. Therefore, the aim of our study was to understand the role of the inflammasome in TAMs and their ensuing capability to release IL-1-like cytokines in the context of lung cancer.

We found that TAMs are another source of IL-1α and IL-1β release in lung cancer lesions in tumor-bearing mice. In particular, the activation of NLRP3 inflammasome via TLR4 signalling led to IL-1β release after caspase-1 activation, whereas, the activation of caspase-11 was important for both IL-1α release and NLRP3-dependent IL-1β release in the lung of tumor-bearing mice.

## RESULTS

### NLRP3 activation leads to IL-1β release from lung tumor-derived macrophages

**Figure 1:**
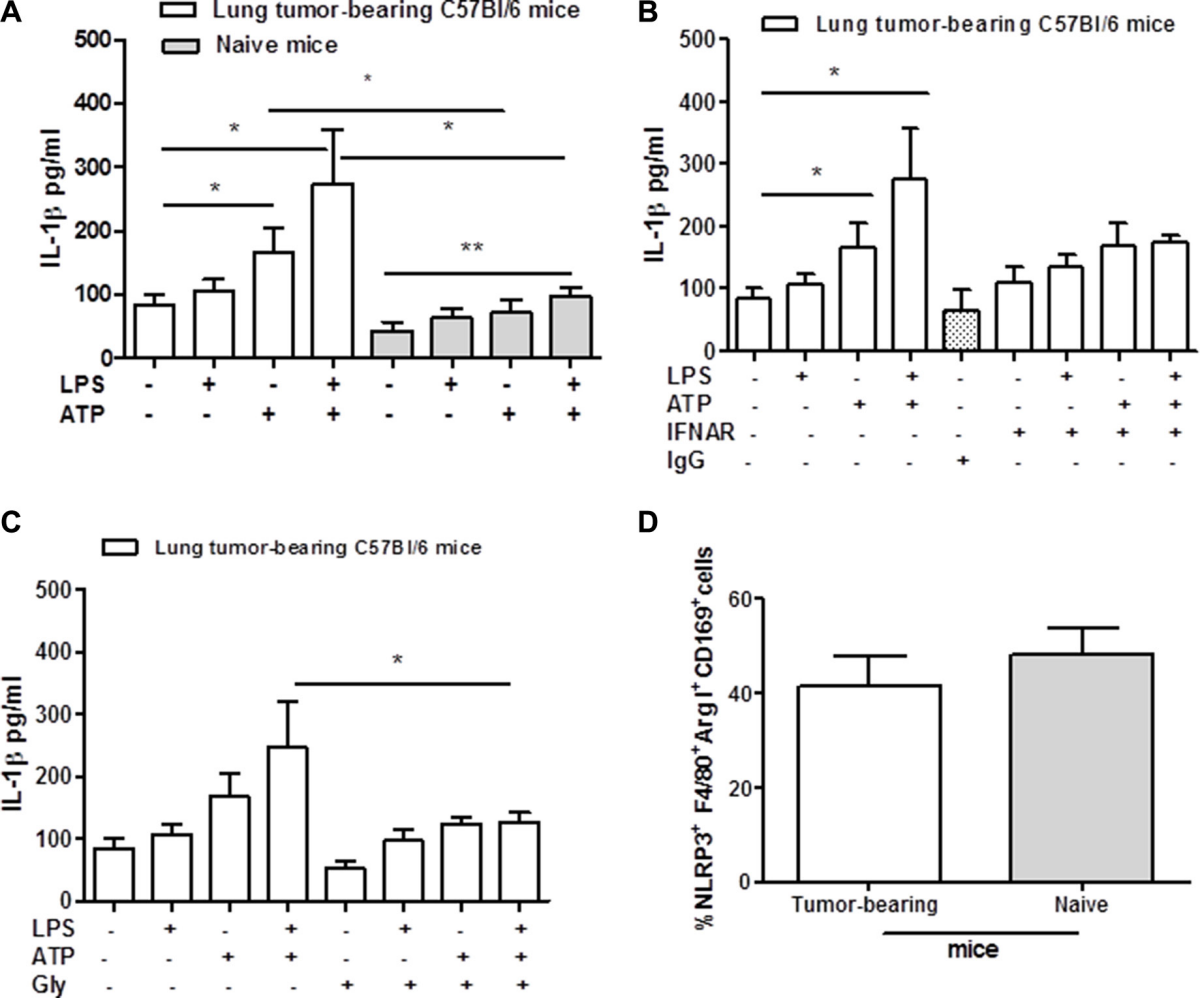
Lung tumor-derived macrophages release IL-1β after NLRP-3 activation Lung TAMs release high levels of IL-1β after ATP and LPS+ATP stimulation compared to naïve lung-derived macrophages (**A**). The addition of LPS+ATP to IFNAR- (**B**) and glybenclamide-treated lung TAMs (**C**) significantly reduced IL-1β levels compared to cells treated with the sole LPS+ATP. (**D**) Flow cytometry analysis shows similar expression of NLRP3 between lung tumor- and naïve lung-derived macrophages. Data represent means ± SEM (*n* = 12). Statistically significant differences are denoted by *and **indicating *p* < 0.05 and *p* < 0.005, respectively, as determined by one-tailed Student's *t* test.

The activation of NLRP3 inflammasome has been widely described in BMDM mimicking the physiological conditions. To understand the role of this complex in lung tumor-associated macrophages (TAMs), we isolated and cultured broncho-alveolar lavage (BAL)-derived cells. Macrophages were stimulated with LPS (0.1 μg/ml) in the presence or not of ATP (0.5 mM) added for 30 minutes. The stimulation of lung TAMs with LPS did not induce the release of IL-1β (Figure 1A), consistent with what already published on the two-signal model of NLRP3 inflammasome activation in BMDM [9]. Similarly, naïve lung-derived macrophages, obtained from non-treated mice, did not release IL-1β after 5 hours of stimulation with LPS (Figure 1A, grey bars). In contrast, the addition of ATP significantly increased the levels of IL-β from lung TAMs (Figure 1A, white bar) compared to naïve lung-derived macrophages (Figure 1A, grey bar). Interestingly, LPS+ATP treatment further increased the levels of IL-1β release from lung TAMs (Figure 1A, white bar). Similarly, naïve lung-derived macrophages released a 2-fold level of IL-1β after LPS + ATP treatment (ctr: 43.15 ± 14.1 vs LPS+ATP: 98.1 ± 12.3, *p* < 0.005). However, these levels were much lower than those observed from LPS + ATP-treated lung TAMs (Figure 1A, white bar vs grey bar), which showed higher basal levels of IL-1β besides the 3-fold increased release (ctr: 84.76 ± 15.65 vs LPS + ATP: 274.6 ± 84.3) than naïve lung-derived macrophages.

To understand whether this effect was type I IFN-dependent, cells were treated with a monoclonal antibody to block type I IFN receptor (IFNAR) [10]. The blockade of IFNAR slightly reduced IL-1β release under LPS+ATP addition, however without reaching a statistical difference (Figure 1B) (*p* = 0.109). The isotype control (IgG) did not alter cytokine levels (Figure 1B).

To prove the involvement of the NLRP3 inflammasome, we treated lung TAMs with glybenclamide (Gly, 1 μM), able to inhibit this complex [11]. The addition of LPS+ATP to glybenclamide-treated cells significantly reduced the levels of IL-1β compared to cells treated with the sole LPS+ATP (Figure 1C). However, we did not observe a complete reduction of IL-1β release in gly+LPS+ATP-treated lung TAMs (Figure 1C). To rule out a potential difference in NLRP3 expression in lung TAMs, we performed a flow cytometry analysis on digested lungs obtained from tumor-bearing mice. Lung tumor-derived macrophages, identified as F4/80^+^Arginase I^+^CD169^+^, had similar expression of NLRP3 as for naïve lung macrophages (Figure 1D).

**Figure 2:**
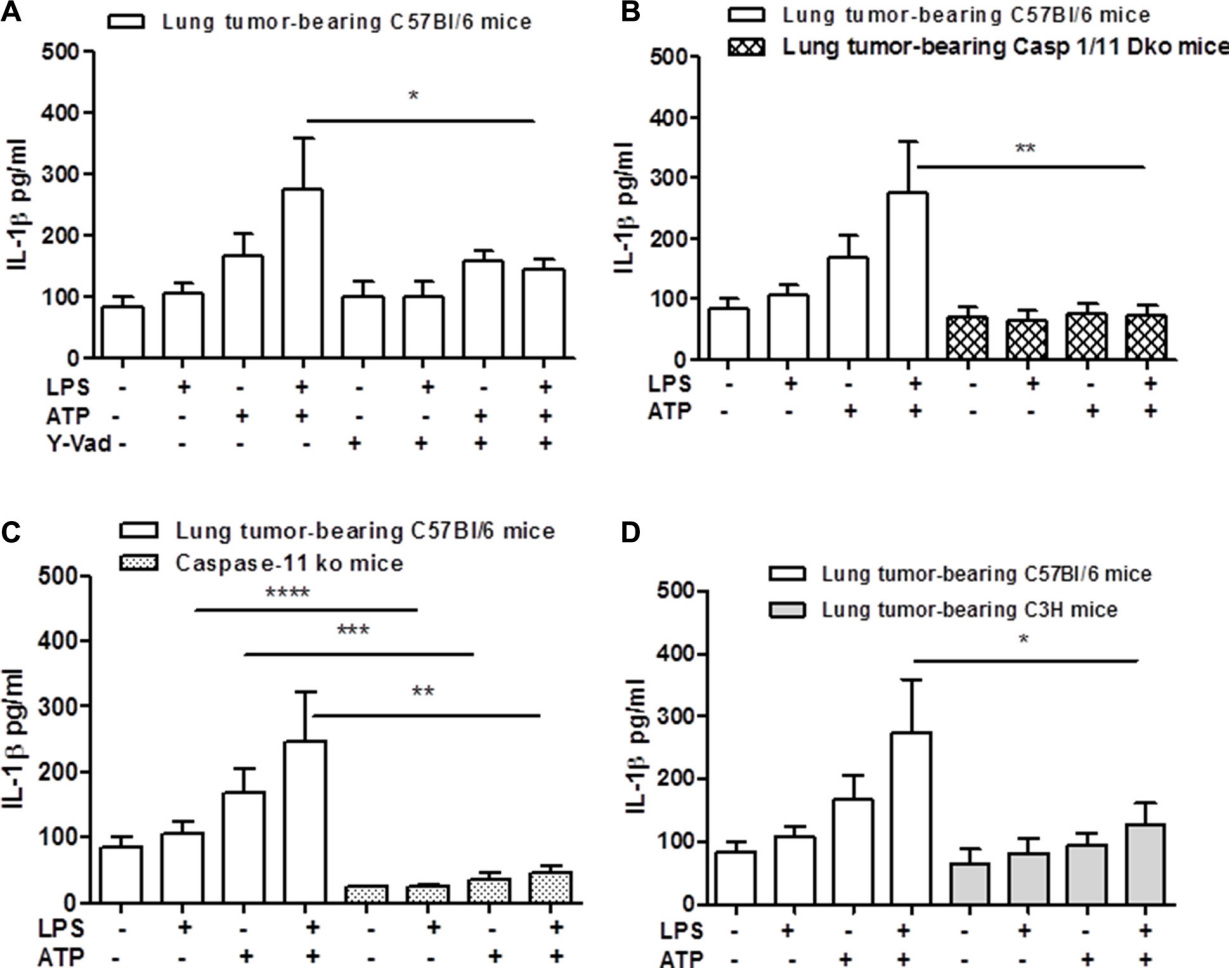
Caspase-1 and caspase-11 modulate IL-1β release after NLRP-3 activation in lung TAMs The inhibition of caspase-1 by means Y-Vad (1 μg/mL) significantly reduced the release of IL-1β after LPS+ATP addition to lung TAMs (**A**). (**B**) The genetic absence of both caspase-1 and caspase-11 or of the sole caspase-11 (**C**) completely abrogated the release of IL-1β from lung TAMs after LPS+ATP stimulation. C3H-derived lung TAMs released reduced levels of IL-1β when LPS+ATP were added (**D**). Data represent means ± SEM (*n* = 12). Statistically significant differences are denoted by *, **, ***and **** indicating *p* < 0.05, *p* < 0.01, *p* < 0.005 and *p* < 0. 001, respectively as determined by Student's *t* test.

Because NLRP3 activation leads to the autocleavage of caspase-1 [12], we went on by pharmacologically inhibiting the active form of caspase-1 by using Ac-Y-VAD-cmk (Y-Vad, 0.1 μg/ml). The inhibition of caspase-1 significantly reduced the release of IL-1β after LPS+ATP addition to lung TAMs (Figure 2A). Moreover, to confirm the involvement of caspase-1 in lung TAM-dependent IL-1β release, we isolated macrophages from the lung of carcinogen-treated caspase-1 knockout (ko) mice. The genetic absence of caspase-1 completely abrogated the release of IL-1β from lung TAMs under LPS+ATP stimulation (Figure 2B).

However, because caspase-11 was described as the intracellular ‘sensor’ of LPS [13] and because caspase-1 ko mice are actually double caspase-1/11 ko mice [14], we used caspase-11 ko mice. The administration of LPS+ATP on cells obtained from carcinogen-exposed caspase-11 ko mice showed a significant reduction of IL-1β release (Figure 2C).

To discriminate the role of TLR4 vs caspase-11 in IL-1β release from lung TAMs, we went on by using C3H mice, which lack a functional TLR4 activity [15]. Lung TAMs from C3H mice showed a complete reduction of IL-1β release after LPS+ATP treatment (Figure 2D). These data implied that TLR4/caspase-1 and caspase-11 axis play a fundamental role for IL-1β release after NLRP3 activation.

**Figure 3:**
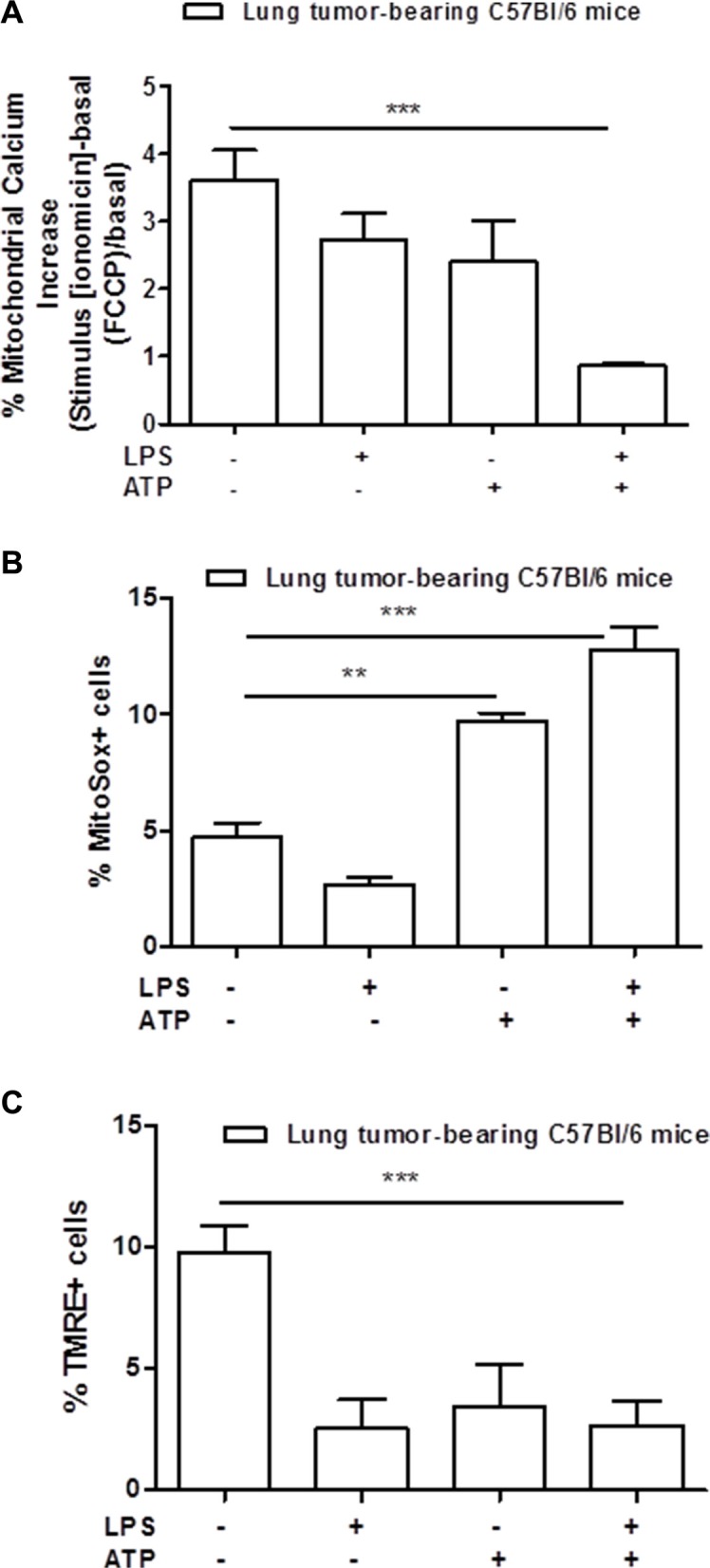
Mitochondria-derived stress is involved in the activation of NLRP3 inflammasome Mitochondrial calcium is significantly released into the cytosol of lung tumor-derived macrophages after LPS+ATP stimulation (**A**). Mitochondrial-derived reactive oxygen species were measured by means of flow cytometry as MitoSox-positive cells (**B**). Lung TAMs show higher positivity to MitoSox after LPS+ATP treatment compared to control (**B**). TMRE assay showed a significant decrease of TMRE-positive cell percentage after LPS+ATP stimulation (**C**). Data represent means ± SEM, (*n* = 12). Statistically significant differences are denoted by **and ***indicating *p* < 0.01 and *p* < 0.005, respectively as determined by Student's *t* test.

Moreover, mitochondria-derived stress has been described as involved in the activation of the NLRP3 inflammasome [16]. Therefore, we measured the levels of calcium released by the mitochondria in lung TAMs. As shown in Figure 3A, the addition of LPS+ATP to lung TAMs did not induce a release of calcium from the mitochondria to the cytoplasm under ionomicin and FCCP stimulation of LPS+ATP-treated cells, implying that the mitochondria were deprived of calcium after stimulation with LPS+ATP. To further confirm the mitochondrial involvement, we analyzed the levels of mitochondrial-derived oxidative stress radicals (mtROS) by means of flow cytometry. The percentage of mitosox positive cells was higher after LPS+ATP treatment compared to control (Figure 3B). Because the release of calcium and of mtDNA implies mitochondrial membrane hyperpolarization [17], we analyzed mitochondrial membrane potential by using the TMRE assay. The percentage of TMRE^+^ cells was significantly lower in lung tumor-derived macrophages treated with LPS+ATP compared to the control (Figure 3C), implying that the TMRE dye was not trapped in the mitochondrial membrane due to depolarization consequent to calcium release.

Taken together these data imply that the activation of NLRP3 in lung TAMs in an mtROS-dependent manner is responsible of caspase-1/caspase-11 activation via the two-signal model that occurs during LPS priming via TLR4 activation.

### Lung tumor-derived macrophages release IL-1α after LPS stimulation

**Figure 4:**
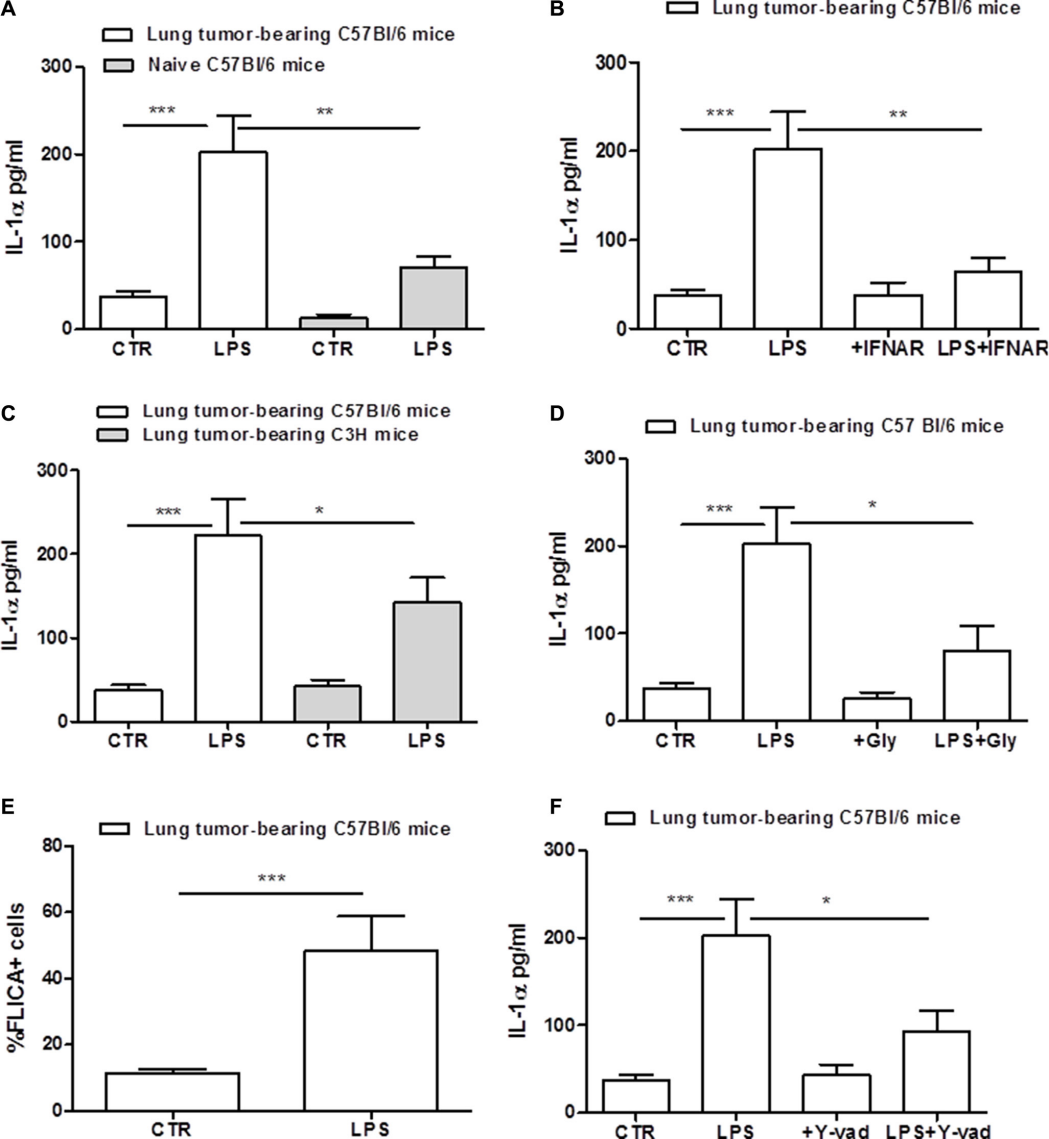
Lung tumor-derived macrophages release IL-1α after LPS stimulation Lung TAMs released higher levels of IL-1α after LPS treatment compared to naïve lung-derived macrophages (**A**). The inhibition of type I IFN receptor (IFNAR) significantly reduced IL-1α release from lung tumor-derived macrophages after LPS stimulation (**B**). C3H-derived TAMs released lower levels of IL-1α levels after LPS treatment (**C**). The addition of LPS to glybenclamide-treated lung TAMs significantly reduced IL-1α levels compared to cells treated with the sole LPS (**D**). Caspase-1 activity was measured by means FAM-FLICA assay kit and expressed according to the absorbance of FLICA^+^ cells; lung tumor-derived macrophages shows high levels of FLICA absorbance, confirming the activity of caspase-1 after LPS administration (**E**). The inhibition of caspase-1 by means Y-Vad (1 μg/mL) significantly reduced the release of IL-1α after LPS addition to lung TAMs (**F**). Data represent means ± SEM, (*n* = 12). Statistically significant differences are denoted by *, **and ***indicating *p* < 0.05, *p* < 0.01 and *p* < 0.005, respectively as determined by Student's *t* test.

IL-1α is another cytokine which release can depend on the inflammasome activation in tumor-associated cells [11]. We observed that the stimulation of cells with the sole LPS induced a significant IL-1α release (Figure 4A). Similarly, lung-derived macrophages obtained from naïve mice released higher levels of IL-1α after LPS addition (Figure 4A, grey bar), although the amount was much lower than that observed in lung TAMs. The inhibition of IFNAR significantly reduced IL-1α release after LPS stimulation of lung TAMs (Figure 4B). To prove the involvement of TLR4 in IL-1α release, we used C3H mice. Differently than IL-1β (Figure 2D), C3H-derived lung TAMs released slightly reduced levels of IL-1α when LPS was added (Figure 4C). To verify the involvement of NLRP3, cells were treated with NLRP3 inhibitor glybenclamide. IL-1α was significantly reduced in LPS+Gly-treated lung TAMs (Figure 4D).

**Figure 5:**
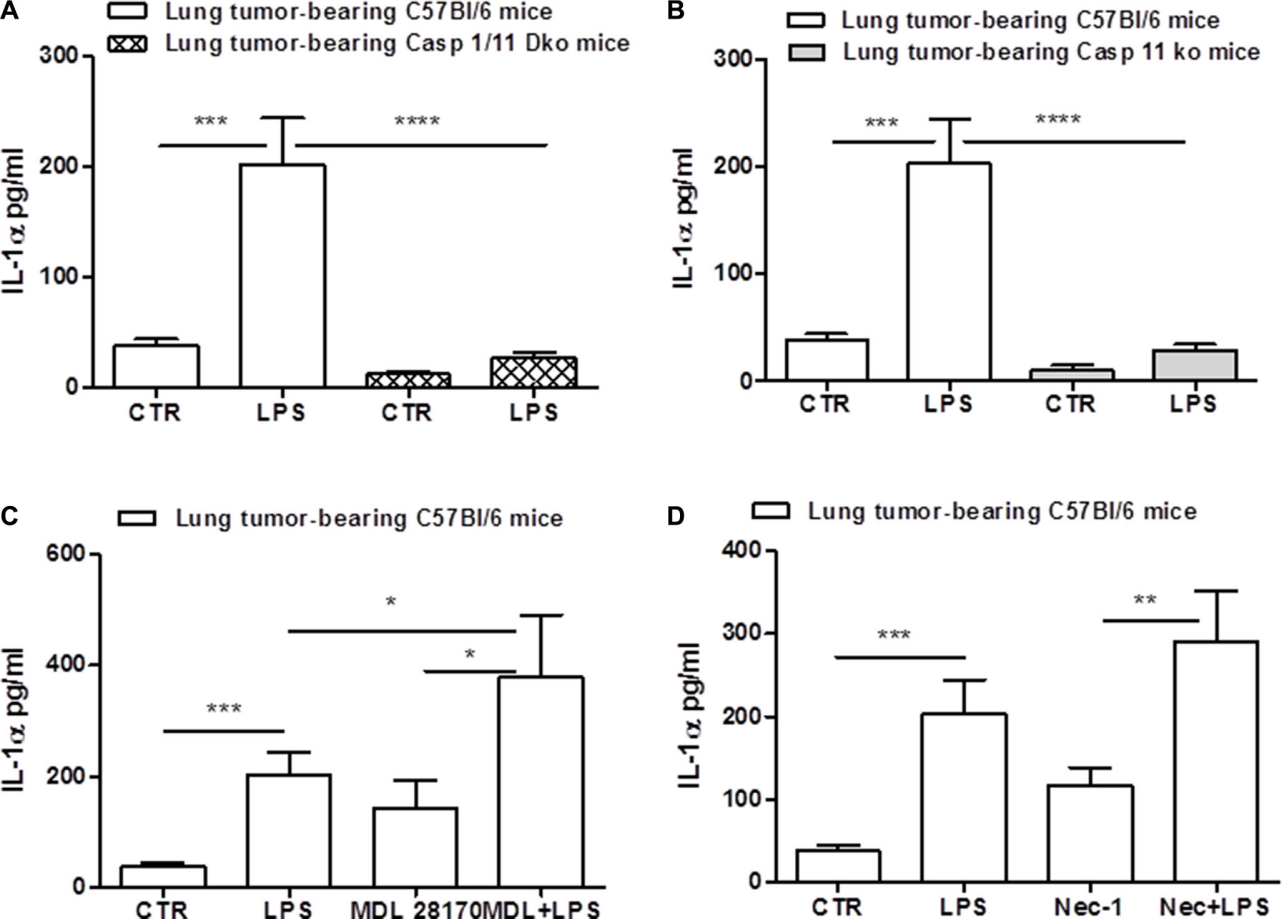
Caspase-11 regulates NLRP3/caspase-1-dependent IL-1α release The genetic absence of both caspase-1 and caspase-11 completely abrogated the release of IL-1α from lung TAMs after LPS stimulation (**A**). Caspase-11 ko lung TAMs released significantly reduced levels of IL-1α when LPS was added (**B**). The inhibition of calpain system by means of MDL 28170 did not reduce IL-1α release after LPS administration (**C**). The administration of Necrostatin-1 did not alter LPS-induced IL-1α release from lung TAMs (**D**). Data represent means ± SEM, (*n* = 12). Statistically significant differences are denoted by *, **, ***and ****indicating *p* < 0.05, *p* < 0.01,*p* < 0.005 and *p* < 0.001, respectively as determined by Student's *t* test.

To verify the role of caspase-1 in this context, we measured the activation of the enzyme by means of flow cytometry. The percentage of FLICA^+^ cells was significantly higher in lung TAMs treated with LPS compared to the control (Figure 4E). To further prove the involvement of caspase-1, we treated cells with Y-Vad, caspase-1 inhibitor. LPS-treated lung TAMs treated with Y-Vad released reduced levels of IL-1α (Figure 4F), although not in a complete manner. To confirm these data, we used caspase-1/caspase-11 double ko mice. Figure 5A shows that the genetic absence of both enzymes completely abrogated IL-1α release in LPS-treated lung TAMs, implying a cross-talk between caspase-1 and caspase-11. However, we observed that caspase-11 ko lung TAMs released significantly reduced levels of IL-1α when LPS was added (Figure 5B), although caspase-1 was still genetically intact, implying that caspase-11 governs over caspase-1 for the release of IL-1α from lung TAMs.

Gross et al. [18], showed that IL-1α was not universally inflammasome-dependent, but rather, calcium-dependent calpain protease activity can lead to IL-1α processing. Therefore, to understand the cross-talk between caspase-1/11-dependent inflammasome and calpain, we inhibited the calpain system by means of MDL 28170. Surprisingly, MDL28170 robustly increased IL-1α release after LPS administration (Figure 5C), implying that the calpain I/II protease can counter inflammasome-dependent IL-1α release from lung TAMs. Moreover, to confirm that IL-1α was not released under cell death, as already observed by the non-alteration of LDH levels (data not shown), we treated lung TAMs with necrostatin-1 to inhibit RIPK1/3-dependent necrosis. The administration of Nec-1 did not alter LPS-induced IL-1α release from lung TAMs (Figure 5D).

Taken together these data imply that while IL-1β release depends on the activation of TLR4 and caspase-11, IL-1α is strictly dependent on caspase-11 which orchestrates NRLP3/caspase-1-dependent inflammasome.

### Adoptive transfer of bone-marrow-derived LPS-primed macrophages increased lung tumor lesions

**Figure 6:**
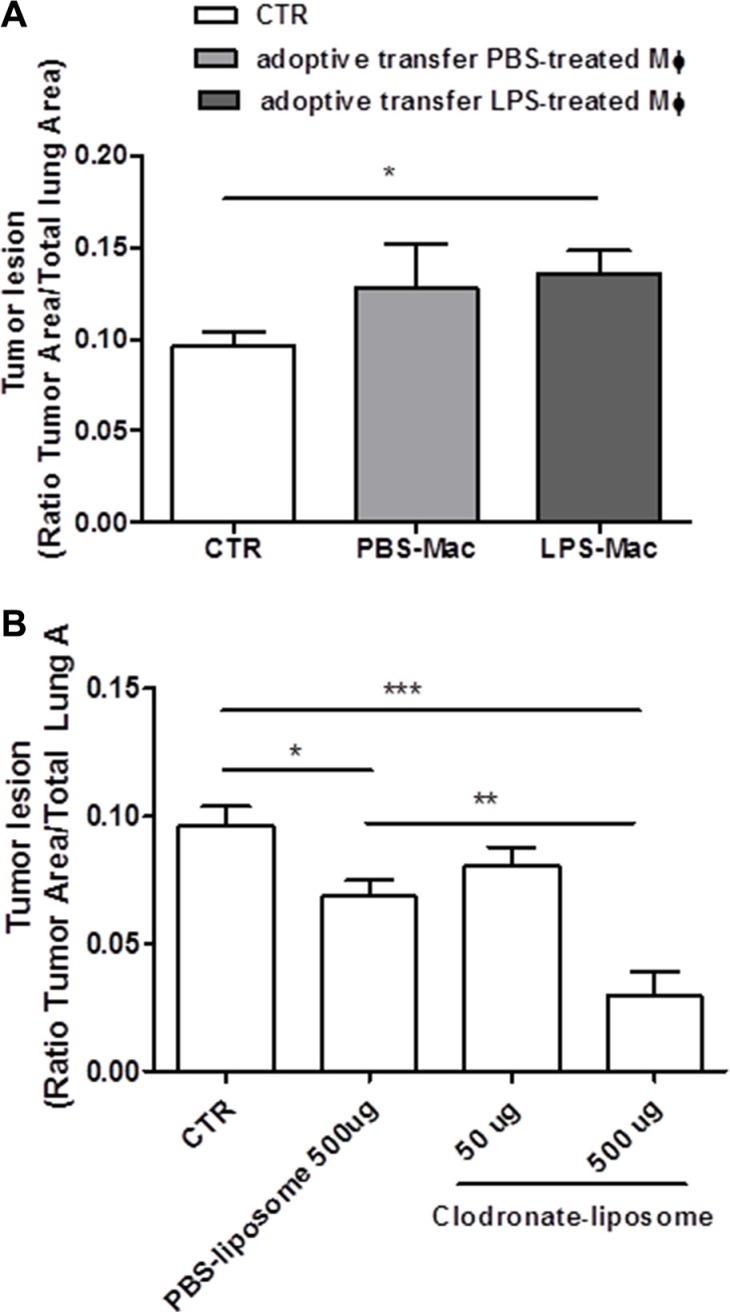
Tumor-associated macrophages are involved in lung tumor formation Bone- marrow-derived macrophages (BMDM) were pulsed for 1 hour with LPS (0.1 μg/ml) or PBS before adoptively transferred into N-methyl-N-nitroso urea (NMU)-exposed C57BL/6 mice; LPS-BMDM inoculation slightly increased tumor lesion in the lung of tumor-bearing mice (**A**). The depletion of macrophages by means of intraperitoneal administration of clodronate-liposome reduced lung tumor formation in a dose-dependent manner (**B**). Data represent means ± SEM, (*n* = 12). Statistically significant differences are denoted by *, **and ***indicating *p* < 0.05, *p* < 0.01, and *p* < 0.005, respectively, as determined by ONE-WAY ANOVA and Student's *t* test.

Our previous data showed that lung TAMs released IL-1-like cytokines under LPS priming which led to the NLRP3 inflammasome activation. To understand the role of the NLRP3 inflammasome in lung TAMs *in vivo*, we went on by performing adoptive transfer experiments of BMDM treated with PBS or LPS (0.1 μg/ml) into carcinogen-exposed C57BL/6 mice. The inoculation of LPS-BMDM slightly increased tumor lesions in the lung (Figure 6A).

To evaluate the immunesuppressive environment, widely associated to tumor progression, we performed flow cytometry analyses to verify the infiltration of myeloid derived suppressor cells (MDSCs) and Treg. The adoptive transfer of LPS-primed macrophages into carcinogen-exposed mice did not alter the percentage of MDSCs (identified as CD11b^+^Gr-1^+^ cells) (Supplementary Figure 2A), but slightly, although not in a significant manner, increased the percentage of Treg (identified as CD4^+^CD25^+^FoxP3^+^ cells) (Supplementary Figure 2B).

### The depletion of tumor-associated macrophages reduced tumor formation in the lung

It is widely described in the literature that TAMs in their M2 phenotype promote tumorigenesis [3]. In order to understand the role of IL-1α- and IL-1β-producing TAMs in lung carcinogenesis, we went on by depleting macrophages by means of clodronate-liposome. The systemic administration of clodronate-liposome reduced lung tumor formation in a dose-dependent manner (Figure 6B). It is to note though that the control PBS-liposomes still had the capability to reduce tumor formation compared to control mice (Figure 6B). However, this effect was not the same as that observed for clodronate-liposome at the dose of 500 μg/mouse (PBS-liposome: 0.0685 ± 0.006 vs Clodronate-liposome: 0.029 ± 0.009).

Taken together these data imply that lung tumor lesions are populated by macrophages which pro-tumor activity is regulated by the activation of the NLRP3 inflammasome that leads to the release of IL-1α and IL-1β.

## DISCUSSION

In this study we found that lung TAMs are IL-1α and IL-1β-producing cells. It is widely recognized that in the tumor microenvironment, IL-1-like cytokines can be secreted by both malignant and infiltrated immune cells [19]. The present work highlights a novel molecular mechanism underlying the suppressive/pro-tumor activity of TAMs during lung carcinogenesis. The activation of caspase-1- and caspase-11-dependent inflammasome related signaling pathways is involved in the release of IL-1β and IL-1α by TAMs. In particular we observed that IL-1β release was dependent on the two-signal model that leads to the NLRP3 inflammasome activation via TLR4 priming; instead, IL-1α release was caspase-11-, but not calpain-, dependent in lung TAMs after LPS treatment.

TAMs are critical components of tumor microenvironment, because they display to tumor growth, tumor angiogenesis, immune suppression, metastasis and chemoresistance [2, 3]. Therefore, their relevance and the modulation of their phenotype is obvious for the success of an anti-tumor immunotherapy especially in the case of such a devastating cancer as lung cancer. TAMs have been described as M2-like cells in that they favor the immunosuppressive environment that leads to tumor progression. Similarly to what already reported in literature, in our experimental conditions, we found that TAMs were arginase I and CD169 positive cells (Supplementary Figure 1B and 1C) as in the case of M2-like phenotype. More importantly, this phenotype was associated to the production of IL-1-like cytokines after the activation of the NLRP3 inflammasome. The inflammasome is a multiprotein complex which biology has been widely studied by using bone-marrow-derived macrophages to mimic a physiological and/or host defense condition [7]. However, it is well established that macrophages tend to assume different phenotype according to the environment they encounter and the tissue they populate. We found that lung TAMs were able to release higher levels of IL-1α and IL-1β than macrophages derived by the lung of naïve mice, implying that the release of IL-1α and IL-1β by TAMs favors lung carcinogenesis. The release of IL-1β has been strictly correlated to the activation of both the canonical (caspase-1-dependent) and non-canonical (caspase-8 and caspase-11-dependent) inflammasome. Here we proved that IL-1β release was caspase-11-, NLRP3/caspase-1-dependent, proving that the activation of the NLRP3 inflammasome in TAMs is pro-tumorigenic. Indeed, the inoculation of LPS-treated macrophages into carcinogen-exposed mice significantly increased tumor formation. On the other hand, the systemic depletion of macrophages by means of clodronate-liposomes reduced lung tumorigenesis. It is to note, though, that TAMs were isolated and then stimulated *in vitro* with LPS ± ATP, not strictly representing what happens *in vivo*. Moreover, the administration of clodronate-liposome depleted all macrophages in the lung without discriminating between IL-1α/β-producing and non-producing cells. Therefore we do not have a direct evidence that IL-1α/β-producing macrophages *in vivo* favor lung tumorigenesis. Though, it is widely known that TAMs facilitate tumorigenesis. In support to our hypothesis, the adoptive transfer of bone-marrow-derived macrophages treated with LPS, which induced the release of IL-1α (data not shown), increased lung tumor lesion. Moreover, it is well known that DAMPs, such as ATP, are responsible of NLRP3 activation [7] as well as it is highly produced in the tumor lesion [20]. In addition, lung TAMs produced higher basal levels of IL-1α and IL-1β than naïve lung macrophages.

IL-1β can facilitate tumor-associated inflammation, rendering the tumor stroma carcinogenic via the release of trophic factors such as FGF2 and VEGF which allow malignant cells, cancer-associated fibroblasts (CAFs) and endothelial cells to fuel and foster tumor cell survival and invasiveness [21]. In addition, IL-1β induces IL-6, which pro-tumorigenic activity is mediated through the activation of STAT-3 in an IL-6 receptor (IL-6R)-dependent manner [22]. In support, in this study we proved that IL-1β-producing TAMs were able to favor lung tumorigenesis after the activation of TLR4/caspase-1 and caspase-11 axis involved in NLRP3 inflammasome. In support, it was already demonstrated that in tumour microenvironment, the stimulation of macrophages with TLR4 ligands can drive towards an M2 phenotype [23].

IL-1α is an alarmin that, differently than IL-1β, is not strictly dependent on caspase-1 but can also be processed by caspase-11 and calpain in the cytoplasm [24–26]. However, we found that lung TAMs stimulated by LPS induced IL-1α release in a caspase-11-, but not calpain-, dependent manner.

IL-1α is one of the predominant cytokine in lung tumor microenvironment. The main source of IL-1α seems to be tumor/epithelial cells. In our recent studies, we observed that this cytokine was also produced by tumor-associated immunesuppressive plasmacytoid dendritic cells (TApDCs) [11]. Similarly, although not in a calpain-dependent manner, we found that lung TAMs can release IL-1α under LPS stimulation, contributing to tumor proliferation.

In conclusion, our study highlights a novel mechanism by which lung tumor lesions are populated by TAMs which produce IL-1α and IL-1β via the activation of the canonical (caspase-1-dependent) and non-canonical (caspase-11-dependent) inflammasome pathways. We believe that sterile alarmins, which nature is still unknown, can lead to NLRP3 activation via TLR4 signalling that leads to IL-1β release after caspase-1 activation, and via the activation of caspase-11, that in turn leads to both NLRP3 activation for IL-1β release and for the release of IL-1α. However, it still remains to elucidate how caspase-11 leads to IL-1α release after LPS recognition. Taken together these data imply that lung tumor lesions are populated by macrophages which pro-tumor activity is regulated by the activation of the NLRP3 inflammasome that leads to the release of IL-1α and IL-1β in a caspase-11/caspase-1-dependent manner.

Our data further increased the knowledge on the inflammasome biology in the tumor microenvironment, pointing at this complex as a promising therapeutic strategy against lung cancer.

## MATERIALS AND METHODS

### Mice

Female specific pathogen-free C57BL/6, caspase-1/11 double knockout (ko), C3H mice (6–8 weeks; Charles River Laboratories, Lecco, Italy) and caspase-11 ko (kindly provided by Dr. Vishva, Genentech, USA) were fed a standard chow diet and housed under specific pathogen-free conditions at the University of Salerno, Department of Pharmacy. All animal experiments were performed under protocols that followed the Italian and European Community Council for Animal Care (2010/63/EU). This study was carried out in strict accordance with the recommendations in the Guide for the Care and Use of Laboratory Animals of the National Institutes of Health. The protocol was approved by the Committee on the Ethics of Animal Experiments of the University of Salerno.

### Experimental protocol

Mice were injected intratracheally (i.t.) with N-methyl-N-nitroso-urea (NMU; Sigma-Aldrich, Rome, Italy) for three consecutive weeks at the dose of 50 μg/mouse (day 0) followed by other two administrations of 10 μg/mouse (day 7 and 14) (Supplementary Figure 1A) [8]. Mice were sacrificed at day 28, four weeks post the first administration of NMU. Broncho-alveolar lavage fluid (BAL) was collected using 0.5 ml of PBS containing 0.5 mM EDTA and cell counts performed.

### Bone-marrow-derived macrophages (BMDM) and adoptive transfer experiments

Mice were anesthetized with isofluorane prior to adoptive transfer experiments. BMDM were previously isolated from the femurs and tibias, and cultured with M-CSF (50 ng/ml; Biosource, CA, USA) for 6-7 days as previously described [27]. Cells were then pulsed for 1 hour with LPS (0.1 μg/ml, ultra pure LPS from *E. coli E0111:B4* Vinci Biochem, Florence, Italy) or PBS before adoptively transferred into mice. Intravenous (i.v.) application of treated or PBS-treated BMDM (5 × 10^5^ cells/mouse) were performed (100 μl) into recipient mice. Inoculation of cells (in PBS), extensively washed, was performed once a week at day 0, 7, 14, 21 before NMU instillation as described in Supplementary Figure 1A. Mice were sacrificed at 28 days. Lungs were isolated and digested with 1U/mL collagenase (Sigma Aldrich, Milan, Italy). Cell suspensions were passed through 70 μm cell strainers, and red blood cells were lysed. Cell suspensions were used for flow cytometric analysis of different cell subtypes.

### Depletion of macrophages *in vivo*

Lung tumor-bearing mice were i.p. treated with PBS liposome or clodronate-liposome (50–500 μg/mouse) (VU Medical Centre, Amsterdam, Netherlands) once a week before the administration of NMU. Mice were sacrificed 28 days post the first exposure to NMU.

### Morphological analysis

Left lung lobes were fixed in OCT medium (Pella Inc., Milan, Italy) and 7 μm cryosections were cut. H&E staining was performed and used to measure the tumour burden. Tumour lesions were analyzed by using serial lung cryosections and expressed as Tumour Lesions/Total Lung Area (in mm^2^) as determined by the ratio of the tumour lesions area compared to the total lung area (Image J Software, NIH, USA).

### Isolation of alveolar macrophages

BAL-derived cells were cultured with RPMI supplemented with 10% FBS, L-Glutamine (2 mM), penicillin (100U/ml) and streptomycin (100 μg/ml) (Sigma-Aldrich, Rome, Italy) in an atmosphere of 5% CO2 at 37°C. Cells were plated for 1 hour to allow macrophages to attach before removing fluttuant cells. Macrophages purity was checked by means of flow cytometry (F4/80^+^CD169^+^Arginase I^+^) and was around 85%, as shown in Supplementary Figure 1B. To verify the phenotype of lung TAMs, we evaluated the expression of arginase I, well-known marker for M2-like macrophages, and iNOS, well-known marker for M1-like macrophages. As shown in Supplementary Figure 1C, the levels of arginase I in lung tumor F4/80^+^ CD169^+^cells were highly increased compared to macrophages in the lung of naïve mice. In contrast, we were not able to detect iNOS levels (data not shown), implying the majority of M2-like cells in the lung of NMU-exposed mice. Then, cells were treated for 5 hours with LPS (0.1 μg/ml), ATP (0.5 mM, for 30 minutes; Sigma Aldrich, Rome, Italy), Glybenclamide (1 μM, Sigma Aldrich, Rome, Italy), Ac-YVAD-cmk (0.1 μg/ml, Sigma Aldrich, Rome, Italy), monoclonal anti-IFNAR antibody (eBioscience, CA, USA).

### Activity of Caspase-1

Caspase-1 activity was measured by means of a commercially available kit FAM FLICA caspase-1 Assay kit (ImmunoChemistry Technologies, USA) and analysed/expressed according to the absorbance (550 nm) of FLICA^+^ cells.

### Flow cytometry analysis

The composition of lung inflammatory cells was determined by flow cytometry (BD FacsCalibur Milan, Italy) using the following antibodies: CD11c-FITC, CD11c-APC, CD11b-PeCy5.5, Gr1-APC, CD3-PeCy5.5, CD4-FITC, CD25-PE, FoxP3-PeCy5.5, F4/80-PE, CD169-FITC, Arg I-PerCp (eBioscience, CA, USA). BAL-derived macrophages were stained for Tetramethyl rhodamine Esther (TMRE, 5 nM, Life Technologies, Monza, Italy) to measure mitochondrial membrane potential alterations. In another set of experiments BAL-derived macrophages were stained for MitoSOX Mitochondrial Superoxide Indicator as indicated in the manufacturer's guide (Life Technologies, USA).

### Cytokine measurements

IL-1α and IL-1β were measured in cell-free supernatant obtained from the BAL-derived macrophages by using commercially available ELISAs (eBioscience, CA, USA).

### Measurement of intracellular calcium (Ca2+)

Intracellular Ca2+concentrations ([Ca2+]i) were measured by using the fluorescent dye Fura 2-AM (Sigma Aldrich, Italy). Lung TAMs (5 × 10^4^ cell/well) were incubated at 37°C with LPS (0.1 μg/ml) for 1 hour and/or ATP (0.5 mM) added for 30 minutes. Thereafter, cells were washed and Fura 2-AM hydrolysis was allowed in calcium free medium, as already reported [11]. Data was expressed as percentage of delta increase of fluorescence ratio (F340/F380 nm) induced by ionomycin (1 μM) or carbonyl cyanide p-trifluoromethoxy-pyhenylhydrazone, (FCCP, 0.05 μM) – basal fluorescence/basal fluorescence ratio (F340/F380 nm).

### Statistical analysis

Results are expressed as means ± SEM. Changes observed in treated groups compared with controls were appropriately analysed using One Way ANOVA, followed by Bonferroni's post-test and/or Student's *t* test. *p* values less than 0.05 were considered significant.

## SUPPLEMENTARY MATERIALS FIGURES



## Abbreviations

TAMs: Tumor-associated macrophages
IL-1: interleukin-1
NLRP3: nod-like receptor 3
