# Supplementary material for: IL-1α and IL-1β-producing macrophages populate lung tumor lesions in mice
Source: Oncotarget. 2016 Aug 12;7(36):58181–92. doi: 10.18632/oncotarget.11276 (PMC5295423; doi:10.18632/oncotarget.11276)
Supplement: Supplementary file 1 [file oncotarget-07-58181-s001.pdf]

IL-1α and IL-1β-producing macrophages populate lung tumor lesions in mice

Supplementary Materials

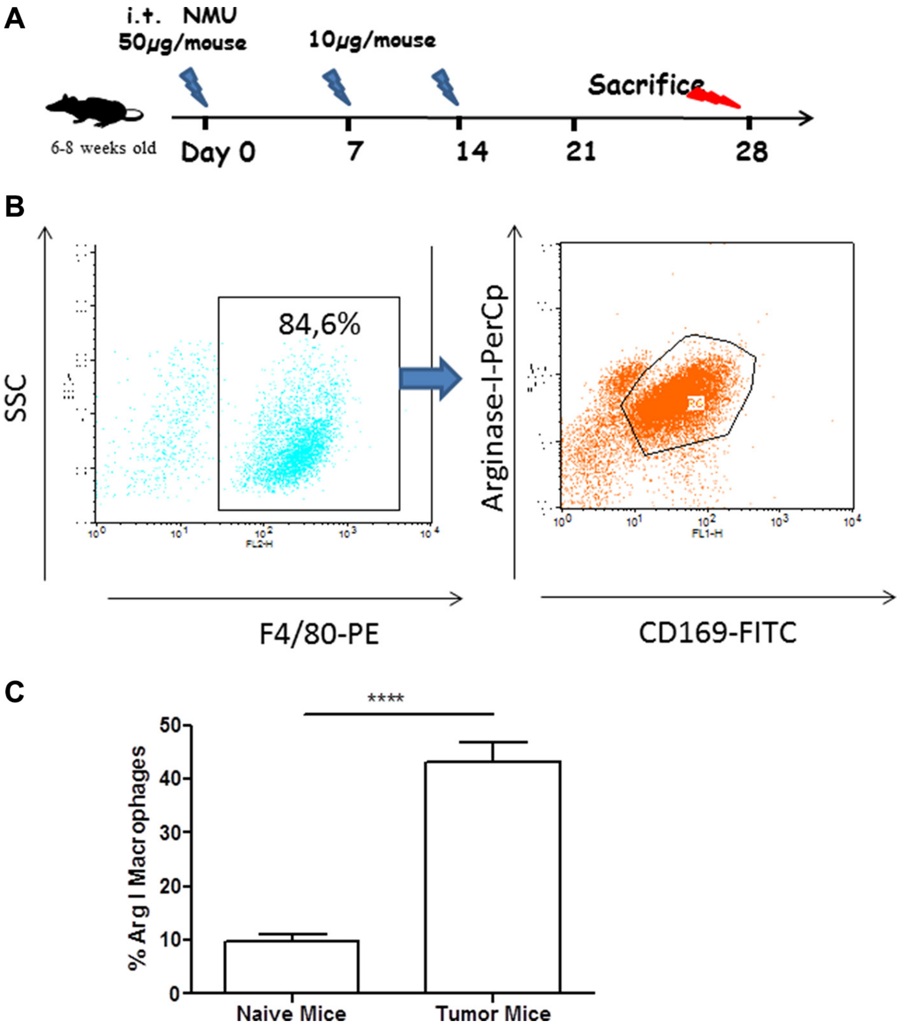

Supplementary Figure S1: (**A**) Experimental protocol. Mice were injected intratracheally (i.t.) with N-methyl-N-nitroso-urea (NMU) for three consecutive weeks at the dose of 50 µg/mouse (day 0) followed by other two administrations of 10 µg/mouse (day 7 and 14). Mice were sacrificed at day 28, four weeks post the first administration of NMU. (**B**) Macrophages were isolated from broncho-alveolar lavage (BAL)-derived cells of naïve and tumor bearing mice; macrophages purity was checked by means of flow cytometry (identified as F4/80+, CD169+ Arginase I+) and was around 85%. (**C**) Arginase I expression is higher in lung tumor- compared to lung naïve-derived macrophages. Data represent means ± SEM, $n = 10$. Statistically significant differences are denoted by ****$p < 0.001$ as determined by Student's t test.

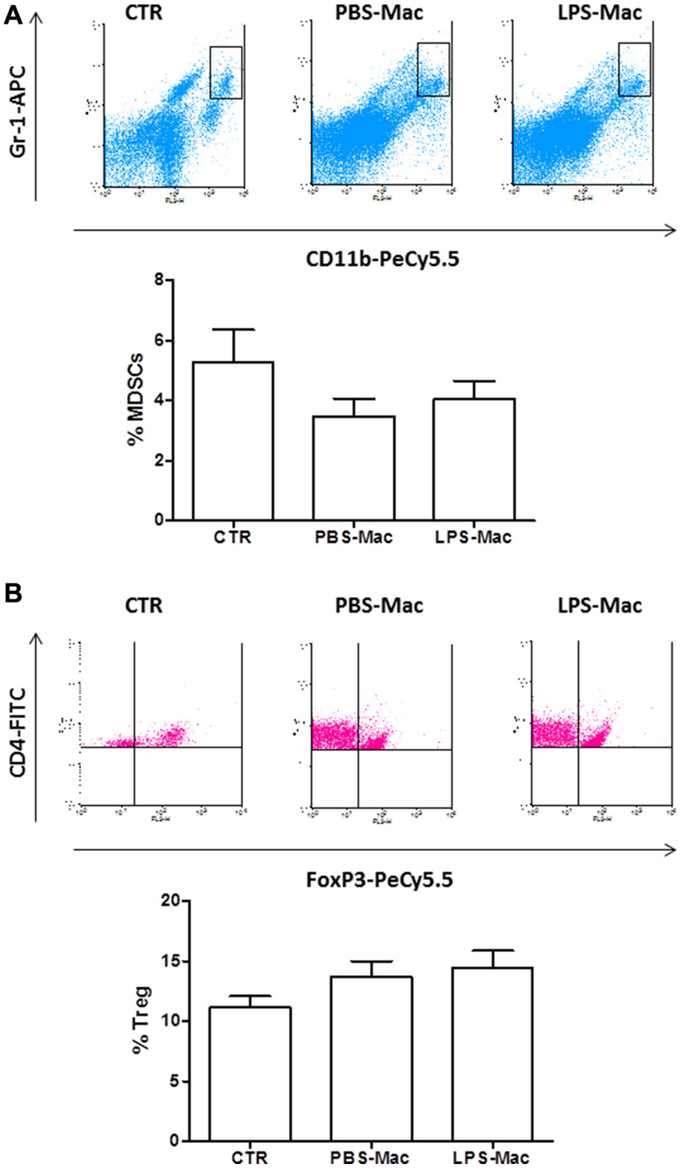

Supplementary Figure S2: The adoptive transfer of LPS-primed macrophages does not alter lung immunesuppressive microenvironment. The adoptive transfer of LPS-primed macrophages into NMU-exposed mice did not alter the percentage of Myeloid-derived suppressor cells (MDSC; identified as CD11b$^+$Gr-1$^+$ cells) (**A**) Representative dot plots are shown), but slightly, although not in a significant manner, increased the percentage of Treg (identified as CD4$^+$CD25$^+$FoxP3$^+$ cells) (**B**). Representative dot plots are shown (B). Data represent means ± SEM, *n* = 10. PBS-Mac = group of mice adoptively transferred with BMDM treated with PBS; LPS-Mac = group of mice adoptively transferred with BMDM treated with LPS.
